# Prolonged puncturing decreases ventriculoperitoneal shunt insertion rate in neonates with posthemorrhagic ventricular dilatation

**DOI:** 10.1007/s00381-026-07408-4

**Published:** 2026-08-01

**Authors:** Dino Gačević, Gerbrich E. van den Bosch, Ronny Knol, Liesbeth S. Smit, Jochem K. H. Spoor

**Affiliations:** 1 Department of Neurosurgery, Erasmus Medical Center, Dr. Molenwaterplein 40, 3015 GD Rotterdam, the Netherlands; 2 Department of Neonatal and Pediatric Intensive Care, Division of Neonatology, Erasmus Medical Center, Sophia Childrens Hospital, Rotterdam, the Netherlands; 3 Department of Neurology, Division of Pediatric Neurology, Sophia Childrens Hospital, Erasmus Medical Center, Rotterdam, the Netherlands

**Keywords:** Hydrocephalus, Newborn, Preterm infant, Ventricular access device

## Abstract

**Purpose:**

Posthemorrhagic ventricular dilatation (PHVD) is a frequent complication of intraventricular hemorrhage (IVH) in preterm infants and is associated with increased neurodevelopmental morbidity. Following national Dutch guidelines, infants with PHVD are generally punctured for up to 28 days via a ventricular access device (VAD), before a ventriculoperitoneal shunt (VPS) is inserted. We aimed to investigate retrospectively whether performing VAD punctures for longer than 28 days decreased the final VPS insertion rate.

**Methods:**

Retrospective single-center cohort study at the Erasmus MC-Sophia Children’s hospital in Rotterdam, the Netherlands. Patients were included if they were born between 2010 and 2020 and treated for PHVD.

**Results:**

Out of 55 included patients, 23 were punctured in low frequency longer than 28 days via their VAD because of improved but persistent ventricular dilatation. Only 14 of them ultimately did require VPS insertion, resulting in a significant reduction of 15% in the final VPS insertion rate (*p* < 0.001).

**Conclusion:**

Puncturing infants with PHVD with a beneficial clinical course via a VAD for longer than 28 days decreases the ventriculoperitoneal shunt insertion rate. Further research on prolonging temporizing punctures and its impact is warranted.

**Supplementary Information:**

The online version contains supplementary material available at 10.1007/s00381-026-07408-4.

## Introduction

Intraventricular hemorrhage (IVH) is the most common neurological complication in preterm infants, affecting roughly 25% of very low birth weight infants [1]. Posthemorrhagic ventricular dilatation (PHVD) is a complication of IVH which develops in 30–50% of patients with a large IVH, due to ventricular obstruction and inflammatory obliteration of the arachnoid granulations by blood depositions [2–4]. About 50% of patients with PHVD develop progressive dilatation which requires removal of cerebrospinal fluid (CSF) [3, 4], as the rise in intracranial pressure can cause deficient myelination and white matter injury [1]. PHVD is associated with neurodevelopmental morbidity, such as cerebral palsy, visuospatial impairment, epilepsy, and cognitive problems [2, 5]. Subsequently, these patients face increased risk of reduced quality of life in comparison to their peers later in life [6]. Therefore, optimal management of PHVD is essential to minimize these risks.

International guidelines for PHVD treatment are lacking, and differences in management between countries are substantial [7]. Our national guideline during the study period recommended early initial CSF extraction by a lumbar puncture (LP) [2]. If this is not possible or insufficient, a ventricular access device (VAD) is inserted, followed by punctures via this device. A VAD is a subcutaneous reservoir that is connected to a tunneled catheter in the lateral ventricle. These two interventions are temporizing, which means that their aim is to allow the infant time to resolve the disbalance in CSF regulation by itself and avoid permanent ventricular shunting. If daily CSF extraction is still necessary after 28 days, a permanent shunt is placed. The most frequently used permanent shunt is the ventriculoperitoneal shunt (VPS).

Permanent shunting of the CSF by VPS is undesirable for several reasons. Once a VPS is placed, a long-term dependence will develop, and the shunt will be necessary for the rest of the patient’s life. VPS placement carries a substantial risk for adverse events, most frequently drain dysfunction and infection [8], and revision rates are substantial [9].

At the time of study, the national guideline recommended VPS insertion after 28 days of VAD puncturing, when patients weigh at least 2000 g, have a CSF erythrocytal count of < 100/mm^3^, and a CSF protein of < 1.5 g/L. A subset of patients shows sufficient regression of ventricular dilation but still needs VAD punctures in low frequency after 28 days of puncturing to maintain this ventricular size. In our institution, the VAD punctures are continued after a period of 28 days in these specific patients, as it is believed that it may omit VPS insertion. However, evidence for this approach is needed. Therefore, the aim of our study was to investigate whether performing VAD punctures for longer than 28 days indeed leads to a lower rate of VPS insertion.

## Methods

We conducted a retrospective single center cohort study at the Erasmus MC-Sophia Children’s Hospital in Rotterdam, the Netherlands.

### Patient selection

Patients were included if they were admitted after birth and treated for PHVD between January 2010 and January 2020 at the level IV neonatal intensive care unit (NICU). Patients with chromosomal disorders or congenital malformations as well as patients with an unusual clinical course (i.e., immediate VPS insertion after traumatic IVH) were excluded.

For the inclusion of patients, a local database of the neonatal neurological admission registry was used. In total, 2338 records were screened for eligibility. Relevant patient characteristics were followed up until a year after birth. These included comorbidities and complications of treatment and duration and quantity of punctures (LP or VAD punctures).

Approval from the research ethics board at the Erasmus MC was acquired (MEC number: MEC-2020-0957). Informed consent of the parents was waived by the ethics board due to the retrospective design of the study.

### Diagnosis and treatment

Diagnosis of PHVD and follow-up of the dilatation was established with cranial ultrasound (cUS). Ventricular size was quantified using Levene’s ventricular index (VI) and Davies’ anterior horn width (AHW) [10–12]. Treatment of PHVD was initiated when the VI surpassed 2 SD + 4 mm or when the ventricles were dilating so acutely that the practitioner deemed it necessary to intervene. First, up to three LPs were applied. If ventricular dilatation persisted or if LPs were not successful, CSF was removed from the ventricles through puncturing of an inserted VAD in regular intervals. At the Erasmus MC, the VAD of choice is the Rickham reservoir (CODMAN® Reservoirs). If PHVD still persisted after 28 days of VAD puncturing, permanent shunting of the CFS by a VPS was indicated. In a subset of patients, the puncturing is extended beyond the period of 28 days, because of the need for VAD punctures in low frequency with regression of ventricular dilation.

### Outcome measures

The primary outcome was the difference in actual VPS insertion rate versus the VPS insertion rate if all patients who received prolonged punctures would have received a VPS at day 28. The secondary outcomes were (1) the complication and revision rate of the VAD among patients who have been punctured via the VAD for > 28 days when compared to ≤ 28 days and (2) the influence of longer puncturing on patient characteristics at VPS insertion (CSF erythrocytal count, CSF protein, and weight at VPS insertion).

### Data collection

Baseline patient characteristics and data respective to the various therapeutic modalities (LP, VAD, and VPS) were collected. Shunt complications based on previous studies included infection, malposition, disconnection, and hemorrhage [8, 13].

### Analysis

Statistical analysis was performed using IBM SPSS Statistics 25.0. Data are presented as median (range) for numerical data and as percentage for categorical data. The *Z*-test, Pearson’s chi-square, or Fisher’s exact test was used to compare categorical data. Data was interpreted with a confidence interval of 95% and was statistically significant at *p* < 0.05.

## Results

**Fig. 1:**
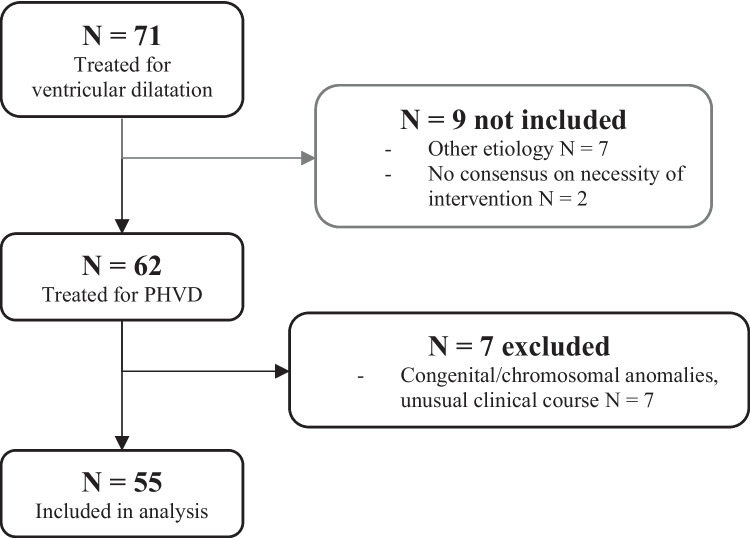
Inclusion flowchart

Between 2010 and 2020, 62 patients received treatment for PHVD at the Erasmus MC. After exclusion of seven patients, 55 were included. The inclusion flowchart is presented in Fig. 1.

The baseline characteristics are presented in Table 1, and the intervention characteristics are presented in Table 2.

**Table 1:** Baseline characteristics. Data presented as median [range] or *N* (valid %)

Parameter	Patients *N* = 55
**Male sex**	32 (58)
**Gestational age at birth, weeks**	30^4/7^ [24^3/7^–40^3/7^]
Preterm (born < 37 weeks of GA)	51 (93)
**Weight at birth, grams**	1495 [690–3650]
**IVH grade**	
I	0 (0)
II	13 (24)
III	17 (30)
Any grade + PVI	25 (45)

*GA*, gestational age; *PVI*, periventricular infarction

**Table 2:** Intervention characteristics. Data presented as median [range] or *N* (valid %)

Parameter	Patients *N* = 55
**Lumbar puncture**	55 (100)
**VAD**	35 (64)
Duration of VAD puncturing, days	34.5 [1–86]
Total VAD punctures	32 [0–119]
**Total duration of puncturing (LP + VAD) days**	28 [1–87]
**VPS**	15 (27)
Time between birth and insertion, days	66 [41–116]
*Characteristics at insertion*	
Weight, grams	2975 [1400–4330]
CSF erythrocytal count*	250 [250–6000]
CSF total protein	1.26 [0.61–4.02]
Complications	6 (40)
Obstruction	1 (7)
Infection	4 (27)
Hematoma	0 (0)
Malpositioning	2 (13)
Dysfunction	2 (13)
Other	0 (0)
Revision	6 (40)
Revision quantity	2 [1–6]

^*^An erythrocytal count of < 500 is averaged out to 250

**Fig. 2:**
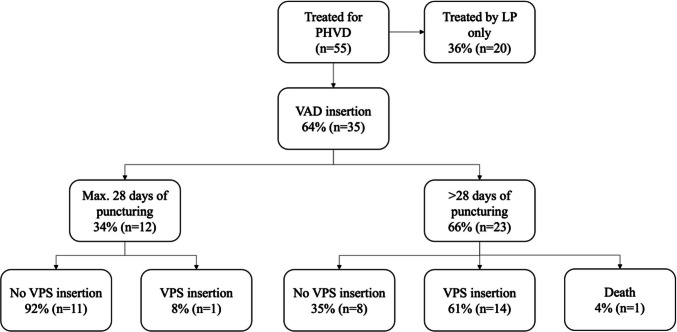
Analysis flowchart

**Fig. 3:**
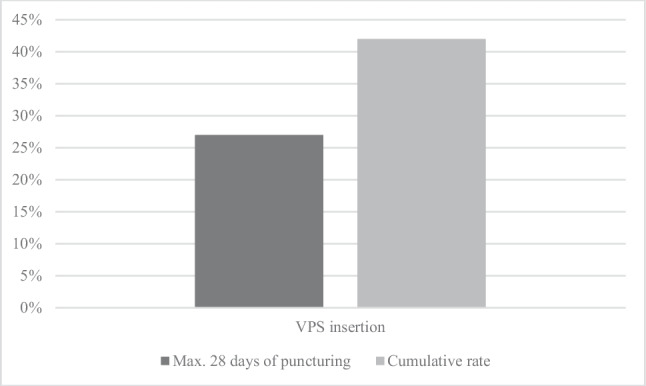
VPS insertion rate diagram

In our cohort, we found a VPS shunt rate of 27% (Table 2). Of the 35 patients in whom a VAD was placed, 23 were punctured for > 28 days. Of these patients, 8 patients underwent no VPS insertion, 14 patients did receive a VPS, and 1 patient died before receiving a VPS (Fig. 2). If all patients who were punctured for > 28 days had received VPS insertion, 23 patients (cumulative rate of 42%) would have received VPS insertion. The difference in this insertion rate and the actual VPS insertion rate is 15% (27% vs. 42%, *χ*^2^ = 28,696, *p* < 0.001) (Fig. 3).

The complication and revision rate of the VAD between the patients who have had punctures for less or more than 28 days did not differ significantly (respectively 25% vs. 26%, *p* = 0.944 and 25% vs. 17%, *p* = 0.596). A comparative analysis is presented in Table 3.

**Table 3:** Intervention characteristics of patients who received VAD punctures for ≤ 28 days vs. > 28 days. Data presented as median [range] or *N* (valid %)

Parameter	Neonates punctured for ≤ 28 days *N* = 12 (34)	Neonates punctured for > 28 days *N* = 23 (66)	*p*-value
**Male sex**	7 (58)	14 (61)	NS
**Gestational age at birth, weeks**	31^2/7^ [25^5/7^–40^0/7^]	30^4/7^ [25^0/7^–40^3/7^]	NS
Preterm (born < 37 weeks of GA)	11 (92)	21 (91)	NS
**Weight at birth, grams**	1732.5 [926–3450]	1470 [755–3610]	NS
**IVH grade**			NS
I	0 (0)	0 (0)	
II	2 (17)	8 (35)	
III	5 (42)	6 (26)	
Any grade + PVI	5 (42)	9 (39)	
**Comorbidities**			
Broncho pulmonary dysplasia	1 (8)	6 (26)	NS
Necrotizing enterocolitis	1 (8)	3 (13)	NS
Sepsis	2 (17)	6 (26)	NS
**Rickhamdrain**			
**Postmenstrual age at placement**	33^1/7^ [28^4/7^–42^5/7^]	33^0/7^ [27^1/7^–45^0/7^]	NS
Length of puncturing, days	11.5 [1–24]	43 [29–86]	** < **0.001
Complications			
Obstruction	0 (0)	1 (4)	NS
Infection	1 (8)	3 (13)	NS
Malpositioning	2 (17)	1 (4)	NS
Impossible to puncture	0 (0)	1 (4)	NS
Other	0 (0)	3 (13)	NS
Revision	3 (25)	4 (17)	NS
Revision quantity	1 [1]	2.5 [1–6]	
**VPS**	1 (8)	14 (61)	** < **0.01
**Died before VPS insertion**	0 (0)	1 (4)	NS

A regression analysis on the association between length of puncturing and patient characteristics before VPS insertion could not be performed due to the low number of patients (*N* = 15) and high variance in results. Among the patients, there was a median weight of 2974 g, CSF protein of 1.26 g/L, and an erythrocytal count of 250.

## Discussion

This study showed that prolonged puncturing via VAD for more than 28 days in a subset of newborns with improved but persisting PHVD decreases the final VPS insertion rate. An overall VPS insertion rate of 27% was recorded among 55 patients treated for PHVD. Eight patients were identified in whom VPS insertion was prevented by puncturing via VAD for longer than 28 days, entailing a significant reduction in the VPS insertion rate of 15%. Meanwhile, puncturing for longer than 28 days did not result in an increase in associated VAD complications or revisions. To our knowledge, no previous research has been conducted on extending the duration of temporizing VAD punctures to lower VPS insertion rate.

In recent years, management of PHVD via temporizing and permanent CSF drainage has focused primarily on the timing of treatment initiation. Early threshold treatment based on the evolution of ventricular dilatation (VI > 97th *p* + 4 mm) leads to a lower VPS insertion rate, less white matter injury, and better neurodevelopmental outcome, when compared to high-threshold treatment, in which treatment starts with the onset of acute symptomatic hydrocephalus [2]. An additional decline in VPS insertion rate was not found by further lowering the treatment threshold to a VI > p97 in an RCT conducted by the ELVIS study group [14]. While post-hoc analysis of this trial showed that patients with a lower treatment threshold did have lower odds of death or severe neurodevelopmental disability when VPS is inserted, the composite adverse outcome between the two intervention groups did not differ statistically significantly [15]. These findings show that further optimalization of treatment in this patient group is a great challenge and it is in this light that this study offers perspective on a readily available, tangible, and cost-effective therapeutic modality, namely by extending the duration of VAD puncturing. However, further research on prolonging temporizing punctures and its impact and safety is warranted.

Our study has several limitations. First, the number of patients included in our study is relatively low. A regression analysis on the length of puncturing and the patient characteristics at VPS insertion could not be performed due to the low number of patients and high variance in results. Accordingly, although differing rates of complication/revision of the VAD were not found in patients that were punctured for more or less than 28 days via the VAD, this finding should be interpreted with caution. Further research should identify whether longer puncturing via the VAD is non-inferior or even beneficial to specific patient groups suffering from PHVD. The retrospective design of the study also potentially limits the accuracy of VPS insertion, complication, and revision rates. These are most likely patients that have had less clinically significant PHVD (i.e., those who may have only received a single LP). Finally, ventricular width is an important parameter for these patients during follow-up, at end of follow-up, and at insertion of any CSF-diverting device. Due to the retrospective nature of this study, we are unable to report reliably on these data. Ventricular width represents an important aspect to include in future (prospective) studies.

In conclusion, this study shows that prolonged puncturing after 28 days in a subset of patients with PHVD via a VAD resulted in a significant decrease in final VPS insertion rate. Additional research on the effect of performing temporizing punctures for an extended period on VPS insertion rates and patient outcome is warranted.

## Supplementary Information

Below is the link to the electronic supplementary material.

ESM 1 DOCX (20.3 KB)

## Data Availability

Generated data is available on request via the corresponding author.
