# Supplementary material for: Prolonged puncturing decreases ventriculoperitoneal shunt insertion rate in neonates with posthemorrhagic ventricular dilatation
Source: Childs Nerv Syst. 2026 Aug 1;42(1):317. doi: 10.1007/s00381-026-07408-4 (PMC13427878; doi:10.1007/s00381-026-07408-4)
Supplement: Supplementary file 1 — DOCX (20.3 KB) [file 381_2026_7408_MOESM1_ESM.docx]

**Supplemental file**

| **Exclusion number** | **Reason of exclusion from study** | **VPS** |
| --- | --- | --- |
| 1 | Antenatal car trauma at AD 28 weeks. Subsequently, no IVH/PHVD was seen on echo. Born at AD 37 weeks, then IVH/PHVD was seen on echo with PVI. VPS insertion immediately because of the serious dilatation. | 1 |
| 2 | Choroidal ateriovenal malformation caused IVH and PHVD. However, she received normal care (LP --> Rickham --> VPS). Possibly, the choroidal arteriovenal malformation has caused a constant bleeding, which may have worsened the outcome of this patient. | 1 |
| 3 | Born at AD 35 weeks. Initially IVH PHVD was treated with LP and rickham drain after which he could go home. Subsequently, after almost a year, progressive dilatation due to liquorcirculatory failure was observed, after which immediate VPS insertion followed. | 1 |
| 4 | Initially, at birth, there were no problems on echo. ECMO caused IVH, alongside many complications. She got ECMO because of low tensions. This caused PHVD, with lumbar punctures and Rickham drain punctures. Also death within first year. | 0 |
| 5 | VPS insertion 273 days after birth because of progressive dilatation withouth symptoms. Rickham punctures administered 25 days before insertion of VPS. | 1 |
| 6 | Plexus choroideus bleeding which caused IVH/PHVD. Born AD 37 weeks. At first, PHVD stabilised. VPS insertion was needed 162 days after birth because of symptoms of PHVD: sunset eyes etc. Non-protocol VPS insertion after only LPs. | 1 |
| 7 | During initial hospital stay, PHVD was not seen. Only mild dilatation maybe due to trauma or rhinovirus infection. Later, symptomatic dilatation was seen in patient. Non-protocol immediate VPS insertion followed. VPS insertion 164 days after birth. Treatment > 28 days of birth | 1 |
